# Multiple network algorithm for epigenetic modules via the integration of genome-wide DNA methylation and gene expression data

**DOI:** 10.1186/s12859-017-1490-6

**Published:** 2017-01-31

**Authors:** Xiaoke Ma, Zaiyi Liu, Zhongyuan Zhang, Xiaotai Huang, Wanxin Tang

**Affiliations:** 1 School of Computer Science and Technology, Xidian University, No.2 South TaiBai Road, Xi’an, People’s Republic of China; 2 Xidian-Ningbo Information Technology Institute, Xidian University, No. 777 Zhongguanxi Road, Ningbo, People’s Republic of China; 3 Department of Radiology, Guangdong General Hospital, Guangdong Academy of Medical Sciences, Zhongshan Road, Guangzhou, People’s Republic of China; 4 School of Statistics and Mathematics, Central University of Finance and Economics, 39 South College Road, Haidian District, Beijing, People’s Republic of China; 5 Department of Nephrology, West China Hospital, Sichuan University, Wuhou District, Chengdu, People’s Republic of China

**Keywords:** Methylation, Network biology, Multiple networks, Epigenetic module

## Abstract

**Background:**

With the increase in the amount of DNA methylation and gene expression data, the epigenetic mechanisms of cancers can be extensively investigate. Available methods integrate the DNA methylation and gene expression data into a network by specifying the anti-correlation between them. However, the correlation between methylation and expression is usually unknown and difficult to determine.

**Results:**

To address this issue, we present a novel multiple network framework for epigenetic modules, namely, **E**pigenetic **M**odule based on **D**ifferential **N**etworks (*EMDN*) algorithm, by simultaneously analyzing DNA methylation and gene expression data. The EMDN algorithm prevents the specification of the correlation between methylation and expression. The accuracy of EMDN algorithm is more efficient than that of modern approaches. On the basis of The Cancer Genome Atlas (TCGA) breast cancer data, we observe that the EMDN algorithm can recognize positively and negatively correlated modules and these modules are significantly more enriched in the known pathways than those obtained by other algorithms. These modules can serve as bio-markers to predict breast cancer subtypes by using methylation profiles, where positively and negatively correlated modules are of equal importance in the classification of cancer subtypes. Epigenetic modules also estimate the survival time of patients, and this factor is critical for cancer therapy.

**Conclusions:**

The proposed model and algorithm provide an effective method for the integrative analysis of DNA methylation and gene expression. The algorithm is freely available as an R-package at https://github.com/william0701/EMDN.

**Electronic supplementary material:**

The online version of this article (doi:10.1186/s12859-017-1490-6) contains supplementary material, which is available to authorized users.

## Background

DNA methylation is a chemical modification of cytosine bases, which is critical for cellular differentiation, cell development and disease progression [1–3]. For example, DNA methylation directly inhibits the binding of transcription factors [4], and methylation aberrations either predispose to or result in disease progression [5]. With biotechnological advancements, DNA methylation is also considered a biomarker of epigenome analysis [6]. Multiple platforms, including reduced representation bisulfite sequencing [7], whole genome shotgun bisulfite sequencing [8], methylC-Seq [9], and target capture bisulfite sequencing [10] and DNA methylation beadarray, have been developed to generate genome-wide DNA methylation data.

High-throughput technologies have generated large-scale genome-wide DNA methylation profiles for various cancers and cell lines, providing great opportunities for revealing the epigenetic mechanisms. Various approaches have been proposed on the basis of methylation profiles to extract DNA methylation patterns. For example, Fleischer et al. [11] identified 18 CpG probes associated with the survival time of breast cancer patients. Hinoue et al. [12] recognized four distinct subgroups in colorectal cancer by analyzing large-scale genome-wide DNA methylation profiles. Yang et al. [13] discovered the comethylation modules across 54 cell lines by using a weighted comethylation network. Varley et al. [14] identified dynamic DNA methylation patterns across 82 human cell lines. Gevaert et al. [15] designed MethylMix to obtain 10 pancancer clusters which reveal a novel similarity across various cancers.

Further details have also been provided [16]. Although DNA methylation patterns have been extensively investigate, open questions have yet to be answered. For example, it is acknowledged that DNA methylation aberrations cause diseases by mediating gene expression [3, 17]. However, how the DNA methylation regulates gene expression remains unknown. Epigenetic modules is critical for revealing the epigenetic mechanisms of cancers. Thus, algorithms for functional epigenetic modules should be established by simultaneously analyzing methylation and gene expression data. However, the design of algorithms for the integrative analysis of DNA methylation and gene expression is highly nontrivial because of two reasons. First, integrative analysis requires large-scale sample matched methylation and gene expression profiles. Second, DNA methylation patterns are difficult to determine because the relationship between methylation and expression is unknown. For instance, it is acknowledged that the correlation between promoter methylation and gene expression is negative. However, the recent evidence indicates that the positive correlation is also presented [14].

Regarding the first issue, the consortium, such as TCGA, has generated sample matched DNA methylation and gene expression data for various cancers [18]. Thus, functional epigenetic module can be identified. Regarding the second issue, researchers developed many algorithms, such as the EpiMod algorithms [19], based on the gene comethylation network by using methylation data and searching modules in the network. However, these algorithms are limited because they are solely based on either methylation or gene expression. Consequently, these algorithms fail to obtain epigenetic modules precisely. To address this problem, researchers developed the FEM algorithm [20] that integrates DNA methylation and gene expression into a protein interaction network by assuming the anti-correlation between DNA methylation and gene expression. The algorithm successfully identifies a novel epigenetically deregulated hotspot and methylated gene modules. This finding indicates the superiority of integrative analysis in terms of revealing methylated gene modules.

Current algorithms are limited by heterogeneous data integrated into a network, and thus require the correlation between DNA methylation and gene expression. However, the correlation between heterogeneous data is unknown and difficult to determine. Recently, multiple networks have been widely used to characterize the complex biological patterns. For example, Ma et al. used multiple networks to characterize the dynamic modules [21]. Cantini et al. proposed a novel multi-network-based strategy to integrate different layers of genomic information and developed algorithm to identify cancer driver genes [22]. Didier et al. assessed aggregation, consensus and multiplex-modularity approaches to detect communities from multiple networks [23]. These algorithms indicate that characterizing biological patterns in multiple networks is more accurate than those in a single network.

**Fig. 1:**
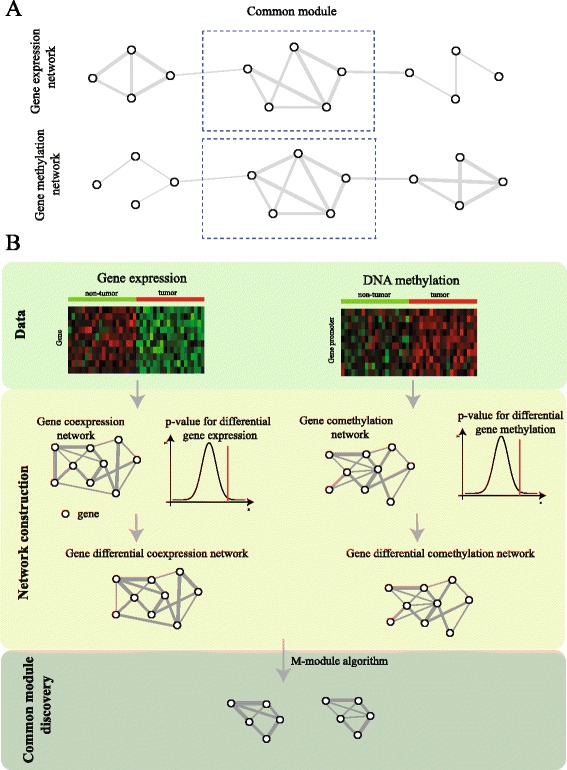
Flowchart of the proposed algorithm. **a** The common modules in both gene expression and methylation networks are defined as the functional epigenetic modules, (**b**) EMDN consists of two major components: differential network construction and functional epigenetic module discovery. The differential network construction consists of two steps: First, the coexpression (comethylation) network is constructed using the gene expression (methylation) profiles, then the p-values of gene expression (methylation) between tumor and non-tumor samples are incorporated into the coexpression (comethylation) network to develop the differential networks. Finally, the algorithm discovers the common modules in multiple differential networks

Therefore, we propose an alternative method that characterizes the epigenetic modules by using multiple networks. We first construct the differential comethylation and coexpression networks, and define the common modules within multiple networks as the epigenetic modules (Fig. 1 a). The proposed strategy is advantageous because it avoids specifying the correlation between heterogeneous data and the accuracy in the identification of epigenetic modules is improved. We also develop the EMDN algorithm by simultaneously analyzing multiple networks (Fig. 1 b). Experimental results demonstrate that the proposed algorithm is more accurate than other algorithms. We further demonstrate that epigenetic modules enriched by the known pathways, serve as biomarkers to predict breast cancer subtypes and survival time of patients. The proposed model and algorithm provide an effective way for the integrative analysis of DNA methylation and gene expression.

## Methods

The EMDN consists of two components: network construction and epigenetic module discovery (Fig. 1 b).

### Step 1: differential network construction

The development of a gene differential coexpression network involves two steps: 1) The development of a binary coexpression network and 2) edge weight assignment based on differential gene expression between normal and tumor conditions. We assume that the gene expression data represents normalized estimates of gene expression intensity and are summarized at the gene level, which includes RNA-seq or expression data generated by using Illumina Beadchip or Affymatrix arrays. The binary gene coexpression network is developed where the edge weight on a pair of genes is the absolute Pearson coefficient of the gene expression profiles. To remove the indirect correlation due to a third gene, w the first order partial Pearson correlation coefficient is used [24]. Finally, edges whose weights are equal or greater than the pre-defined threshold *δ* are selected.

Using the Limma package [25], the *p*-values of the gene expression difference between normal and tumor samples for each gene are obtained. The *p*-values are corrected by Benjamini-Hochberg (BH) [26]. Then, the gene differential coexpression network is developed by using the *p*-values. Specifically, the weight *w*_*ij*_ on edge (*i*,*j*) in the differential network is defined as

1 $$ w_{ij} = \left\{ \begin{array}{ll} \frac{\left(\left|\log P_{i}+\log P_{j}\right|\right)^{1/2}}{\left(2\max_{l}\left|\log P_{l}\right|\right)^{1/2}}& \mathrm{if~ } |cor(i,j)|\geq \delta,\\ 0 & \mathrm{if~ } |cor(i,j)|< \delta, \end{array} \right.  $$

where *P*_*i*_ is the *p*-value of the differential expression for gene *i*, and *c* *o* *r*(*i*,*j*) is the value of Pearson correlation between the *i*-th and *j*-th gene based on the expression profiles. The genes that are co-expressed and significantly differentially expressed are assumed to have assigned heavy weights. In this study, the *δ* is 0.4. This value leads to the maximal number of genes connected in all the networks.

Analogously, the differential comethylation network is developed using the gene methylation profiles.

### Step 2: discovering the epigenetic modules

To infer the epigenetic modules, EMDN is based our recently developed *M-module* algorithm that is designed for identifying common modules in multiple molecular interaction networks [21]. EMDN consists of three steps: seed prioritization, module search by seed expansion and refinement of candidate modules.

The seed prioritization ranks genes in multiple networks by using the topological feature of the gene in the networks. Specifically, for each network *G*_*k*_ with the weighted adjacency matrix *W*_*k*_=(*w*_*ijk*_)_*n*×*n*_, a function *f*_*k*_:*V*→*R* is developed, where *f*_*k*_(*i*) denotes the importance of the *i*-th gene in the corresponding network. The function is defined as

2 $$ f_{k} = \alpha A^{'}_{k}f_{k}+(1-\alpha)Y,  $$

where $A^{'}_{k}f_{k}$ denotes the topological importance of nodes and *Y* is the vector for the prior information. The parameter *α* controls the relative weight of the topological importance and prior knowledge. $A^{'}_{k}$ is normalized adjacency matrix, i.e., $A^{'}_{k}=D^{-1/2}_{k}W_{k}D^{-1/2}_{k}$ with *D*_*k*_=*d* *i* *a* *g*(*d*_1*k*_,*d*_2*k*_,…,*d*_*nk*_). We use the following iteration-based algorithm to obtain *f*_*k*_:

3 $$ f^{[t+1]}_{k} = \alpha A^{'}_{k}f^{[t]}_{k}+(1-\alpha)Y,  $$

where *t* denotes the iteration, and $f^{[0]}_{k}=0$. No prior information is used. Thus, we set *Y*=0. The iteration is terminated if there is no change between *f* ^[*t*+1]^ and *f* ^[*t*]^. Usually, only 20 iterations are observed. We calculate the z-score for *f*_*k*_. After ranking the genes in all networks, the ranks of genes are obtained in multiple networks, denoted by *R*=[*f*_1_,*f*_2_,…,*f*_*M*_]. The final gene ranking is obtained by using the z-score of genes in multiple networks, i.e., the row sum of *R*.

Starting with each seed, the module search step iteratively adds genes whose addition causes the maximum decrease in the graph entropy-based objective function until no decrease in the objective function is observed. Given a module *C*, the entropy function for network *G*_*k*_ is defined as

4 $$ H_{k}(C) = -p_{k}\log p_{k}-(1-p_{k})\log (1-p_{k}),  $$

where $p_{k}=\sum _{i,j\in C}w_{ijk}/\sum _{i\in C}w_{ijk}$. The entropy function for *C* across all networks is defined as

5 $$ H(C) = \sum_{k=1}^{2}H_{k}(C)/|C|.  $$

The module search procedure expands the module by using Eq. (5). Each time the gene whose addition leads to the maximum decrease of *H*(*C*) is included in *C* until no gene can improve *H*(*C*).

During the refinement step, candidate modules whose sizes are smaller than 5 are removed. To merge overlapping function modules, two modules whose Jaccard index is greater than 0.5 are merged.

### Data

We download gene expression and DNA methylation data for breast cancer from TCGA database. Specifically, 869 samples with matched level-3 Illumina 450k methylation data and HiSeq RSEM gene-normalized RNA-seq data are obtained with 785 tumor and 84 normal samples. For methylation data, the *β* signal of the probe is used, which is calculated as the methylated signal divided by the sum of the methylated and unmethylated signal. For the RNA-seq data, the reads per kilobase of exon model per million mapped reads (RPKM) is used. The clinical information is also obtained from TCGA. In all these datasets, probes with more than 30% missing values are removed, and probes with less than 30% missing values are imputed using the R package PAMR [27].

### Gene methylation profiles

To assign DNA methylation to a given gene (for Illumina 450k data), we follow the strategy in the Ref. [20]. Specifically, the average value of the probes mapping within 200 bp of the transcription start site (TSS) is assigned to the gene. If no probes mapped within 200 bp of the TSS, we use the average value of probes mapping to the 1st exon of the gene. If such probes are also not available, we use the average value of probes mapping within 1500 bp of the TSS.

### Eigengenes of modules

The eigengene of a module is defined as the first principal component based on singular value decomposition (SVD) [28]. In details, the gene expression matrix of a given module is denoted by *X*=(*x*_*ij*_) where the index *i* corresponds to the module genes and the index *j* corresponds to the samples. The singular value decomposition of *X* is denoted by

6 $$ X=UDV^{T},  $$

where the columns of the matrices *U* and *V* are the left- and right-singular vectors, respectively. The first column of *V* is the module expression eigengene. Similarly, we obtain the module methylation eigengene by the gene methylation profiles.

### Correlation of module methylation and expression

The correlation between module methylation and expression is defined as the Pearson correlation coefficient between module methylation and methylation eigengenes. The correlation is significant when BH correction is used with the cutoff 0.05. If the sign of Pearson coefficient is greater than 0, a positive correlation is observed, and negative if otherwise.

### Survival analysis

We use the function *coxph* (R package *survival*) [29] to implement the Cox proportional hazard model to analyze the association of methylation profile of each epigenetic module with the patient survival.

We use the prognostic index to generate high- and low-risk patient groups

7 $$ index_{i} = \sum_{c =1}^{k}\beta_{c}X_{ci}  $$

where *k* is the number of cancer-specific modules, *β*_*c*_ is the regression coefficient of the Cox proportional hazard model for the *c*-th module and *X*_*ci*_ is the average methylation level of genes within the *c*-th module in the *i*-th patient. Patients are grouped into high- and low-risk groups based on the median of prognostic index. The survival difference between these two groups of patients is obtained by using the Kaplan-Meier estimator and log-rank method.

### HAND2 module and simulation

As the true module, we select the *HAND2* module (Fig. 3 b), since the biological and clinical significance of the driver gene, *HAND2*, has been extensively validated [17]. To fully assess the sensitivity and specificity of the proposed method, the simulation model of HAND2 module is also adapted [20], in which it simulates statistics of differential methylation and differential expression on the protein interaction network. The model bootstrapped statistics for the member genes of this module to come from the top and lower 5% statistics quartiles, with the statistics of the rest of the network genes bootstrapped from the middle 90% portion. For each simulation run, the accuracy is recorded.

### Statistical significance of modules

The statistical significance of modules is based on the null score distribution of the random modules generated using randomized networks. Each network is completely randomized 100 times by degree-preserved edge shuffling. To construct the null distribution, we perform EMDN on the randomized networks. The empirical *P*-value of module is calculated as the probability of the random module having the observed score or greater by chance. *P*-values are corrected for multiple testing using BH correction. The cutoff of *P*-value is 0.05, and it is statistically considered.

### Functional analysis of epigenetic modules

To assess the functional relevance of the epigenetic modules, we perform the gene ontology (GO) enrichment analysis by using the hypergeometric test. We obtain enrichment lists with BH corrected *p*-value (cutoff 0.05).

### Features of SVM on functional epigenetic modules

Given a module *C*, we obtain the methylation level of each gene across all patient samples [30], denoted by *X*_*ij*_ for the *i*-th gene and *j*-th patient. For each sample *j*, the activity score of the *k*-th module is defined as the average gene methylation of all genes within the module, i.e.,

8 $$ X_{C} = \sum_{i\in C}X_{ij}/\sqrt{|C|}.  $$

where |*C*| is the number of genes in module *C*. A feature vector is constructed for each module.

### Simulated multiple networks

The simulated networks were generated following and the true classification of genes into clusters is known [31]. We simulated three networks, each of which has 256 nodes. In each network there are eight clusters of equal size. The parameter controls the noise level of a network by controlling the ratio of intra-cluster edges to inter-cluster edges that are connected to a node. The degree of each node is fixed to 32. As mixed parameter increases from 0 to 0.6, the detection of clusters in the networks becomes increasingly difficult. The multiple networks contain two artificial networks, where the one network with noise level 0.1 and the other with noise level from 0.1 to 0.6.

### Normalized mutual information

The normalized mutual information (NMI) [32] is based on the confusion matrix *N* whose rows correspond to the real modules in standard partition *P* ^∗^ and the columns correspond to the modules in obtained partition *P*. The element *N*_*ij*_ is the number of vertices overlapped by the *i*-th real and *j*-th predicted module. The NMI is defined as

9 $$ {}NMI\left(P,P^{*}\right)=\frac{-2\sum_{i=1}^{|P|}\sum_{j=1}^{|P^{*}|}N_{ij} \log\left(\frac{N_{ij}N}{N_{i.}N_{.j}}\right)} {\sum_{i=1}^{|P|}N_{i.}\log\left(\frac{N_{i.}}{N}\right)+\sum_{i=1}^{|P^{*}|}N_{.j}\log\left(\frac{N_{.j}}{N}\right)},  $$

where |*P*| is the number of module in *P* and *N*_*i*._ is the sum of the *i*-th row of matrix.

## Results and discussion

### Performance benchmarking of algorithms on artificial networks

For a comparative analysis, three algorithms are selected, including Consensus clustering (CSC) [22], multiple-modularity method (MolTi) [23] and spectral clustering (SPEC) [33]. The SPEC method is not suitable for multiple networks. Therefore, the method is extended by using the consensus strategy [22].

**Fig. 2:**
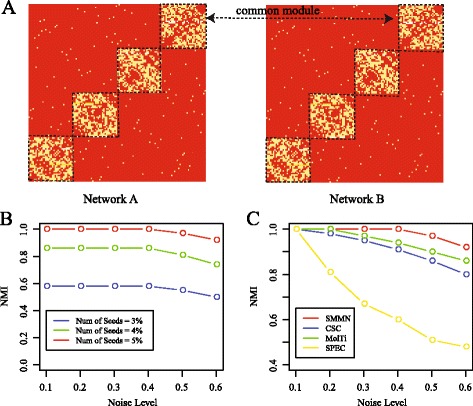
Performance of various algorithms on the artificial networks (**a**) Heatmap of artificial multiple networks with noise level 0.1, (**b**) The number of seeds that affects the performance of EMDN, and (**c**) Performance of the compared algorithms on the artificial networks with various noise levels

These algorithms are used in the artificial networks to test the performance. Figure 2 a is the heatmap of multiple networks where the common modules are surrounded by the dashed line. We used the normalized mutual information (NMI) as a measure to quantify the performance. Before giving the performance of the compared algorithms, we first investigate how the parameter affects the performance of EMDN. The only parameter involved is the number of seeds. The result is illustrated in Fig. 2 b, where the number of seeds increased from 3 to 5%, and the accuracy increases dramatically. The result indicates that 5% is the optimal value. The accuracy of various algorithms on the artificial networks is shown in Fig. 2 c, where EMDN outperforms the others and the MolTi algorithm is better than the CSC and SPEC methods.

**Fig. 3:**
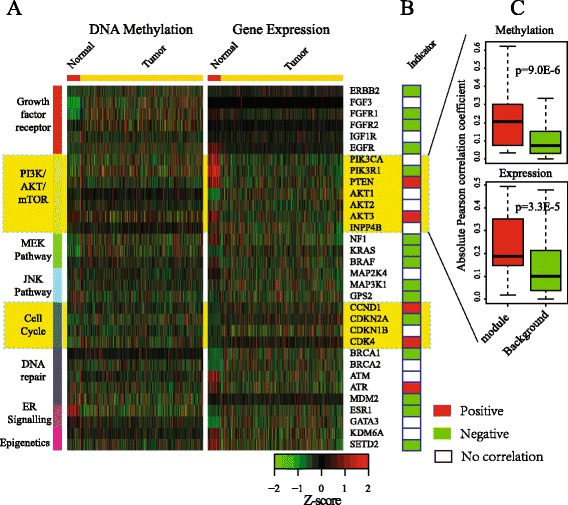
The correlation between the methylation and expression of genes and modules is associated with breast cancer metastasis. **a** Heatmaps of genes and pathways between normal and tumor samples for DNA methylation (*left*) and gene expression (*right*), (**b**) Indicator for correlation between the methylation and expression, where *red* denotes positively correlated, *green* for a negative correlation and white for noncorrelation, and (**c**) Comparison between Pairwise correlation coefficient among modules and random pathways based on methylation (*top*) and gene expression (*bottom*)

### Negatively and positively correlated genes and pathways are common in breast cancer

Prior to giving the performance of EMDN algorithm, we first investigate the existence of positively correlated genes and pathways for breast cancer. We select the acknowledged genes and pathways associated with breast cancer metastasis from Ref. [34], including 32 genes and 8 pathways as shown in Fig. 3 a. The correlation between gene promoter methylation and gene expression is presented in Fig. 3 b, where 15 genes are negatively correlated (green), 5 positively correlated (red) and 12 uncorrelated (white). The result is consistent with that presented in Ref. [11], where the positively correlated genes are also important in breast cancer metastasis. Moreover, the pathways can be categorized into two groups: pathways only with negatively correlated genes as well as pathways mixed with both positively and negatively correlated genes. For example, the MEK and growth factor receptor pathways are the first group, whereas the PI3K/AKT/mTOR and cell cycle are classified under the second group. Moreover, we find that the PI3K/AKT/mTOR and cell cycle are significantly positively correlated (Material Section). It is reasonable because majority of genes within pathway are positively correlated, for example, the coefficients are 0.12, –0.11, and 0.375 for CCND1, CDKN2A, and CDK4, respectively. The result indicates the existence of positively correlated genes and pathways for breast cancer, which is also the main focus of this paper.

**Fig. 4:**
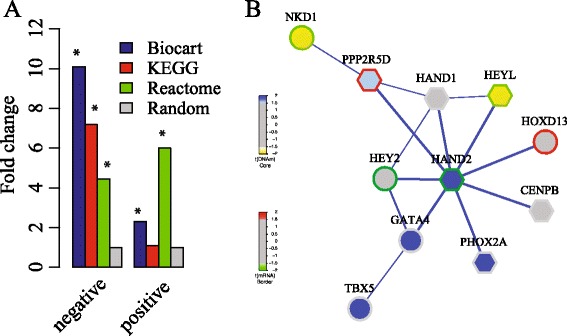
Pathway expression and methylation correlation. **a** The fold change of pathways in various databases in terms of correlation between gene expression and methylation, where ∗ denotes a significant difference when compared to the random modules (*p*-value < 0.05, Fisher exact test), and (**b**) The HAND2 modules, where *Node* colours denote the differential DNA methylation statistics as indicated. *Border* colors denote the differential expression statistics. Hexagon genes are recognized by EMDN

Whether the positively and negatively correlated pathways are ubiquitous for breast cancer in various databases, including the KEGG [35], Reactome [36], Biocart [37]. We find that the pathways in all databases are both negatively and positively correlated although the majority of the pathways are negatively correlated (Additional file 1: Figure S2). Specifically, 26.7% of the pathways in BioCart (21.4% in KEGG, and 16.5% in Reactome) are negatively correlated with coefficient ≤ –0.1 (*p*-value=5E-3, Cor.test), whereas 6.5% of the pathways in Biocart (3.1% in KEGG, and 4.7% in Reactome) are positively correlated with coefficient ≥ 0.1. To check the significance of the percentage of correlated pathways, we compare the fold change of pathway percentage whose absolute value of correlation coefficient is equal or greater than 0.1 with the size-matched random pathways (Fig. 4 a). The fold changes are 10.1 (BioCart), 7.2 (KEGG), and 4.5 (REACTOME) for negatively correlated pathways, whereas the fold changes are 2.3 (BioCart), 1.2 (KEGG), 6.0 (REACTOME) for postively correlated pathways. Both positively and negatively correlated pathways are significantly higher than the random modules (Fisher exact test, *p*-value <0.05).

The gene level correlation between methylation and expression profiles is also calculated. As shown in Additional file 2: Figure S1A, the gene level methylation-expression correlations are both positive and negative. For example, 5678 genes with coefficients less than –0.1 are observed, whereas only 1096 genes with coefficients greater than 0.1 are observed.

### Performance benchmarking of algorithms on *HAND2* module

To evaluate the performance of EMDN algorithm, we compare it with several state-of-the-art, including Consensus clustering (CSC) [22], multiple-modularity method (MolTi) [23], FEM [20], EpiMod algorithm [19]. Because EpiMod is designed for single network, we applied it to differential comethylation and coexpression network respectively, denoted by EpiMod-Meth and EpiMod-Exp.

We employed the *HAND2* module as benchmark, which has 11 genes centered at HAND2 gene (Fig. 4 b). The EpiMod-Meth and EpiMod-Exp algorithms cannot discover the module, while the rest algorithms discover the module. The result demonstrates that the integrative analysis of methylation and expression data is promising for functional epigenetic modules. The module recognized by EMDN contains 8 genes, in which 6 genes are from HAND2 module (hexagon nodes in Fig. 4 b, accuracy =54.5%), whereas the accuracy of FEM, CSC and MolTi is 37.9%, 22.9% and 17.4%. The reason why the FEM and EMDN algorithms outperform CSC and MolTi is that both CSS and MolTi are not designed for epigenetic modules. The results indicates that multiple networks based strategy is more effective than the single network based approaches.

To fully characterize the performance, we compare FEM and EMDN on the simulated HAND2 module (Material Section) by using the accuracy (ACC), positive predictive value (PPV), false discovery rate (FDR) and false positive rate (FPR). The comparison between FEM and EMDN is in Table 1. EMDN is better than FEM on ACC, FPR, PPV as well as FDR. Two possible reasons are presented to explain why EMDN is better than FEM. The first reason is that the multiple network model is a better way to characterize the functional epigenetic module than the single network based integration strategy because it avoids specifying the correlation between gene expression and methylation. The second reason is that the module search strategy used in EMDN is effective.

**Table 1:** The accuracy of compared algorithms on the simulated network, where values with bold font are the best performance for each column among algorithms

	ACC	FPR	PPV	FDR
FEM	37.9%	0.003	0.379	0.621
EMDN	54.5%	0.0003	0.750	0.125

### Performance benchmarking of EMDN on TCGA breast cancer networks

Next, we compare the algorithms using a compendium of 869 samples from TCGA. The differential comethylation (coexpression) network has 12,142 genes and 11516060 (4939426) edges. We identify 26, 16, 19 and 17 modules using EMDN, EpiMod-Exp, EpiMod-Meth and EMDN, respectively.

**Fig. 5:**
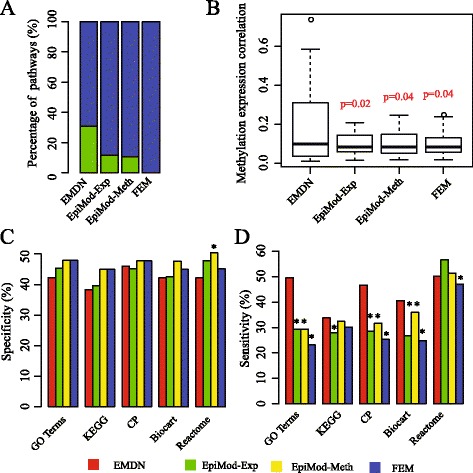
Performance benchmarking on TCGA data. **a** Percentage of negatively and positively correlated modules, where green denotes a positive correlation and blue for negative correlation, (**b**) Distribution of absolute values of module correlation between methylation and expression, where *p*-values are computed using the student t-test, (**c**) Specificity of the methods. Gene modules found by each method are evaluated by a set of gold-standard pathway annotation. Specificity is defined as the fraction of gene modules significantly enriched for genes of some reference sets, and (**d**) Sensitivity of the methods. Sensitivity is defined as the fraction of reference sets significantly enriched for genes of some modules. Enrichment *p*-values are computed using hypergeometric test and corrected by Benjamin-Hochberg, where * denotes *p*<0.05

No gold-standard exists for epigenetic modules in breast cancer. Thus, we compare the algorithms from three aspects. First, the correlation coefficients between the methylation and expression of the modules are used to validate the performance because the ultimate goal of the algorithms is to discover methylated gene modules. The higher the coefficient is, the more likely the methylated module is. We compare the absolute value of correlation coefficients for epigenetic modules obtained each algorithm as shown in Fig. 5 b. We conclude that the coefficients of the modules obtained by EMDN are significantly higher than those obtained by other algorithms (EMDN vs EpiMod-Exp *p*-value=0.02, EMDN vs EpiMod-Meth *p*-value=0.04, EMDN vs FEM *p*-value=0.04, Student t-test). The result demonstrate that the proposed algorithm is more effective in discovering methylated gene modules.

Second, both positively and negatively correlated modules for breast cancer are shown. Moreover, whether all these algorithms can discover both negatively and positively correlated modules is questioned. The percentages of positively and negatively correlated modules for each algorithm are shown in Fig. 5 a. We conclude that only FEM algorithm cannot recognize positively correlated modules. The reason is that FEM assumes the negative correlation between gene expression and methylation.

Finally, we check how well the modules are enriched in the known pathways. Multiple reference pathway annotations are used, including GO [38], KEGG [35], Reactome [36], Biocart [37], and canonical pathways from MSigDB [39]. To evaluate the performance, we use specificity and sensitivity to quantify the accuracy, as shown in Fig. 5 c and d. Based on the results, we conclude that EMDN achieves significantly higher specificity when evaluated using all reference sets while a comparable sensitivity is maintained (*p*-value < 0.05, one-sided Fisher’s exact test).

### Epigenetic modules serve as biomarkers that improve the accuracy of breast cancer subtype prediction

**Fig. 6:**
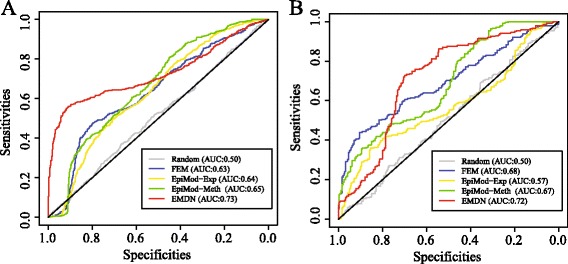
Epigenetic modules as biomarkers for tumor prediction. **a** Classification of TCGA data. ROC curves for predicting breast cancer tumor using different feature sets, including random modules (RG, modules obtained by FEM-DM, FEM, EpiMod-Exp as well as EpiMod-Meth, (**b**) Validation of prediction on external data. The classification model is trained on TCGA data and tested on external data

The genes or pathways has been used to improve the prognosis of breast cancer [11, 40]. We hypothesize that the epigenetic modules may also likely be considered as biomarkers that predict luminal A/B, HER2 and Basal-like breast cancer subtypes. We use the support vector machine (SVM) package for multiple classes classification [41]. As a baseline comparison, we first compare the classification accuracy using the following feature sets: modules obtained by various algorithms and size-matched set of random modules. SVM is used for classification by using methylation profiles (Methods), where the area under curve (AUC) is employed to measure performance. The results on TCGA data using fivefold cross validation are shown in Fig. 6 a, suggesting that the modules obtained by EMDN are more discriminative than others on breast cancer subtype prediction. The EMDN had significantly higher AUC (0.73 vs 0.65, *p*-value=4.1E-4, Delong ROC test [42]).

To check the possibilities that the above result is biased because of parameter selection, we perform additional analysis by varying each of these parameters. The result is consistent when we employ a 3/10-fold cross validation (Additional file 3: Figure S3A and B). To check out the possibility that the confounding factors in TCGA dataset contribute to the classification accuracy, we evaluate the performance of the SVM classifiers (trained on TCGA data) using an external data (GSE60185, Illumina 450k, 285 samples) [11]. The result indicates that the similar tendency is consistent with that in the TCGA dataset as shown in Fig. 6 b. The results indicate that the performance is not due to the hidden confounding factors in the TCGA data (0.72 vs 0.68, *P*=7.9E-3, Delong ROC test).

Finally, we investigate the possible reasons why EMDN outperforms FEM. We quantify the importance of each epigenetic module, which is defined as the AUC of SVM based on the single module for subtype prediction. We compare the feature importance between positively and negatively correlated modules. There is no significant difference between the positively and negatively correlated modules (Additional file 3: Figure S3C, *p*-value=0.3, Student t-test). Both of positive and negative correlation epigenetic modules are discriminative for cancer tumor prediction.

### Epigenetic modules are associated with survival time of patients

Genes and modules are associated with patient survival time in breast cancer [11]. We hypothesize that the epigenetic modules are also associated with the clinical outcome using gene methylation(expression). The multivariable Cox proportional hazard models are used to predict the survival time (Materials Section).

**Fig. 7:**
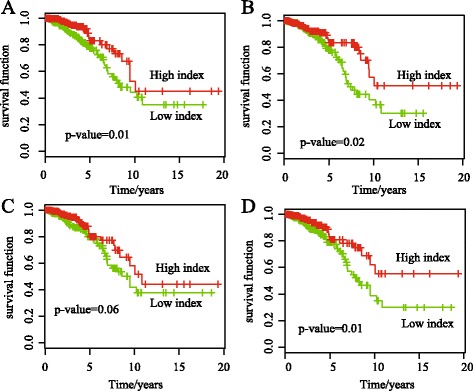
Kaplan-Meier survival analysis for patients based on the epigenetic modules. Negatively correlated modules for survival time of patients: (**a**) Module 1 with a *p*-value of 0.01 obtained using the log-rank test, (**b**) Module 8 with a *p*-value of 0.02. Positively correlated modules for survival time of patients: (**c**) Module 7 with a *p*-value of 0.06, (**d**) Module 11 with a *p*-value of 0.01

For each module, we predict the patient survival time by using the methylation (expression) and the patients are segregated well into high- and low-risk groups according to the patient survival time. We find that both the positively and negatively correlated modules divide the patients into two groups whose survival time is significant different (*p*-value <0.05, Fig. 7). For example, The module 1 and 8 are negatively correlated and the patients are divided into two groups with significantly different survival time (Fig. 7 a and b). The module 7 and 11 are positively correlated and the patients are divided into two groups with significantly different survival time (Fig. 7 c and d). In module 7, the enriched GO term is *immune response* (*p*-value=1.7E-4, hypergeometric Test) with genes *FCER1A*, *CD1B*, *CD1A* and *MS4A2*, etc. The results further demonstrated that both the positively and negatively correlated modules are critical for patient survival analysis.

Moreover, seven out of 26 modules obtained by EMDN are significantly associated with the survival time of patients, whereas 112 out of 1107 random modules with similar sizes are significantly associated with survival time of patients (*p*-value=1.4E-2, Fisher Exact test).

### Comparison between coexpression/comethylation networks and differential networks

It is necessary to validate the possibility to replace the differential networks with the coexpression and comethylation networks for EMDN. We identify 21 modules by using the EMDN algorithm based on the coexpression and comethylation networks (co-exp/meth modules). We first check the module methylation expression correlation coefficients between co-exp/meth modules and differential modules. The differential modules have significantly higher coefficients than the co-exp/meth modules (*p*-value=3.6E-4, Student t-test, Additional file 4: Figure S4A). Then, we check the sensitivity and specificity of the two groups of modules, showing that the differential modules are more enriched by the known pathways than the co-exp/meth modules (Fisher exact test, *p*-value < 0.05, Additional file 4: Figure S4B and C). Finally, we compare the two groups of modules on breast cancer subtype prediction, and the result demonstrates that the differential modules are more discriminative than the co-exp/meth modules (*p*-value =6.7E-5, Delong test, Additional file 4: Figure S4D). The result indicates that the differential network is preferred than coexpression (comethylation) network.

## Conclusions

Exploring the functional epigenetic patterns is critical for understanding the mechanisms of biological processes. Recent technology has made it possible to perform simultaneously multi-platform genomic profiling of biological samples, including DNA methylation and gene expression. However, the systematic and integrative analysis of heterogeneous data for discovering biologically relevant patterns is currently scarce.

Currently, all the available methods integrate the methylation and expression data into a scaffold network, such as protein interaction, which requires specifying the correlation between gene expression and methylation. However, the specification of the correlation is unreasonable because there are both positive and negative correlation. In this study, a novel strategy is presented to characterize functional epigenetic modules by using heterogeneous differential networks. The functional epigenetic module discovery corresponds to find common modules in differential coexpression and comethylation networks. Overall, EMDN possesses several unique advantages. (i) It provides a novel strategy for the integrative analysis of methylation and expression data. (ii) It is more effective and accurate than modern methods because correlation specification is not required. (iii) The method is easy to extend for other data, such as Illumina 27k methylation, and Chip-seq.

The basic concept of EMDN should be modified in future work. First, the algorithm is a generalized framework to any cohort of expression and methylation data, although this study uses breast cancer as a proof-of-principle. Second, data integration (e.g., epigenome and CNVs) might further enhance the identification of complicated molecular events involved in heterogeneous data.

## Additional files

Additional file 1 Supplementary Figure S2. Distribution of pathway methylation and expression correlation coefficients for various databases: (A) negatively correlated pathways, and (B) positively correlated pathways. (EPS 529 kb)

Additional file 2 Supplementary Figure S1. Positively and negatively correlated genes in breast cancer between methylation and expression: (A) distribution of positively and negatively correlated coefficients, and (B) pie chart for positively and negatively correlated genes. (EPS 661 kb)

Additional file 3 Supplementary Figure S3. Breast cancer subtype prediction: (A) accuracy of compared algorithms using a 3-fold cross validation, (B) accuracy of compared algorithms using a 10-fold cross validation, and (C) feature importance of positively and negatively correlated modules in classifying the breast cancer subtypes. (EPS 839 kb)

Additional file 4 Supplementary Figure S4. Comparison between coexpression/methylation and differential networks: (A) distributions of correlation coefficients of modules, (B) accuracy on classifying the breast cancer subtypes, (C) specificity of modules on the enrichment of known pathways, and (D) sensitivity of modules on the enrichment of known pathways. (EPS 802 kb)

## Abbreviations

HAND2: Heart- and neural crest derivatives-expressed protein 2
KEGG: Kyoto encyclopedia of genes and genomes
SVD: Singular value decomposition
SVM: Support vector machine. TCGA: The cancer genome atlas - cancer genome
TSS: Transcription start site
